# Developing an animal model for Alzheimer's disease with Lewybodies using the Fischer TG344‐AD rat model

**DOI:** 10.1002/alz70855_106772

**Published:** 2025-12-25

**Authors:** Matthew Mandrozos, Tina Beckett, Mary Hill, Jessica Ribeiro, Nareh Tahmasian, Joanne Mclaurin

**Affiliations:** ^1^ University of Toronto, Toronto, ON, Canada; ^2^ Sunnybrook Research Institute, Toronto, ON, Canada

## Abstract

**Background:**

Alzheimer's disease (AD) pathology is characterized by the presence of two aberrantly folded proteins. Amyloid‐β (Aβ) peptide, in the form of extracellular plaques and tau which forms intracellular inclusions. Increasingly, pathological studies have identified the presence of additional misfolded proteins, associated with other neurodegenerative disease, in AD patient brains. It is speculated that the presence of these co‐pathologies contributes to the observed heterogeneity in AD. The most frequent co‐pathology is Lewy Bodies (LBs), more commonly associated with Parkinson's disease, found primarily in the amygdala of AD brains. Patients with the Lewy Body Variant of AD (LBV‐AD) are found to have an accelerated disease course compared to AD patients without LB pathology. This study aims to characterize a novel preclinical rat model that can recapitulate the main pathological hallmarks of LBV‐AD.

**Method:**

Unilateral stereotaxic injection of AAV 2/5 serotype virus, containing either full length (hSNCA) or truncated (SYN119) human α‐synuclein, was performed on sex‐balanced F344TgAD and non‐transgenic littermate rats at 9‐months of age. Cohorts were aged‐out to 3‐, 5‐ and 9‐ month end‐points, at which brain tissue was collected. Histology was then performed on tissue sections to quantify pathological load and markers of neurodegeneration.

**Result:**

Over‐expression of both hSNCA and SYN119 produced α‐synuclein aggregates within the amygdala and striatum at 3‐months post‐injection (3 m.p.i.) within the injected hemisphere. Initial pathological analysis of 3 m.p.i. cohort showed a trend to increased aggregate positive cells in transgenic versus nontransgenic rats. At 5 m.p.i. this trend had reversed with significantly more LB pathology in the non‐transgenic hSNCA injected rats compared to Tg hSNCA injected and both Tg and NTg injected SYN119 groups.

**Conclusion:**

Both hSNCA and SYN119 produced α‐synuclein within the amygdala and striatum at 3 m.p.i., which increased with time after injection.

